# 3,4-Dihydroxybenzaldehyde Exerts Anti-Alzheimer’s Effects by Inhibiting Aβ Protofibril Assembly and Activating Antioxidant Defense Mechanisms

**DOI:** 10.3390/ijms27031599

**Published:** 2026-02-06

**Authors:** Zhourong Zhao, Lin Yang, Zhuo Zhang, Jia Song, Chao Zhang, Xiaohua Duan

**Affiliations:** Yunnan Key Laboratory of Dai and Yi Medicines, Yunnan University of Chinese Medicine, Kunming 650500, China; zhaozr_cpu@163.com (Z.Z.);

**Keywords:** Alzheimer’s disease, *Caenorhabditis elegans*, 3,4-dihydroxybenzaldehyde, Aβ protofibrils, antioxidant

## Abstract

3,4-Dihydroxybenzaldehyde (DBD) is a polyphenolic active constituent derived from *Gastrodia elata*. Its characteristic phenolic structure is associated with diverse bioactivities, such as anti-inflammatory, antioxidant, and cardioprotective effects. However, its role and underlying mechanisms in combating Alzheimer’s disease (AD) remain inadequately elucidated. In this study, we employed computational and experimental approaches to investigate the anti-AD effects of DBD. Molecular dynamics simulations revealed that DBD binds to Aβ fibrils via π–π stacking, hydrophobic interactions, and hydrogen bonds, suggesting its potential to disrupt Aβ fibril stability and thereby inhibit aggregation. In vivo experiments in an AD C. elegans model demonstrated that 2 mM DBD treatment significantly delayed paralysis and extended lifespan. It also improved locomotor activity and pharyngeal pumping rates, while reducing lipofuscin accumulation. These results collectively suggest that DBD promotes healthspan-associated phenotypes. Broad-targeted metabolomics analysis indicated that DBD significantly altered the metabolic profile of the worms. Further mechanistic investigations suggested that the protective effects of DBD are associated with the activation of the DAF-16/FOXO and SKN-1/Nrf2 signaling pathways, accompanied by enhanced resistance to oxidative and thermal stress in nematodes. These findings suggest that DBD exhibits anti-AD potential through multimodal mechanisms, which involve interference with Aβ toxicity and reinforcement of cellular defense. This study supports DBD as a candidate compound and provides a rationale for its further investigation.

## 1. Introduction

Alzheimer’s disease (AD) is a progressive neurodegenerative disorder characterized by cognitive decline and behavioral changes. Its primary pathological hallmarks are the deposition of extracellular β-amyloid (Aβ) plaques and the formation of intracellular neurofibrillary tangles (NFTs) composed of hyperphosphorylated tau protein [1]. Presently, around 55 million people globally are affected by Alzheimer’s, with estimates suggesting a rise to 152 million by 2050 [2]. In the clinic, AD is treated mainly with cholinesterase inhibitors such as donepezil and galantamine, as well as N-methyl-D-aspartate (NMDA) receptor antagonists such as amantadine, which are all focused on symptomatic improvement and lack efficacy in improving the course of the disease. The development of AD is intricate, characterized by multiple interacting factors. Key hypotheses include the amyloid cascade hypothesis, tau pathology, cholinergic dysfunction, neuroinflammation, and oxidative stress [3], with abnormal Aβ accumulation considered a primary pathogenic factor. The enzymatic cleavage of the amyloid precursor protein (APP) by β-secretases leads to the production of Aβ peptides. Aβ’s hydrophobicity leads to its aggregation into neurotoxic oligomers and protofibrils with β-sheet structures, eventually forming amyloid plaques [4]. Since its introduction, the amyloid hypothesis has represented one of the core theories explaining the pathological mechanisms behind Alzheimer’s disease. The classic “amyloid cascade hypothesis” primarily emphasizes the deposition of extracellular insoluble fibrillar plaques and their physical disruptive effects. Currently, soluble oligomers (AβO) generated during Aβ aggregation prior to plaque formation are considered the more neurotoxic species. These oligomers can directly impair synaptic function and induce oxidative stress and inflammatory responses, leading to neuronal dysfunction and death. Recent advances in cryo-electron microscopy (cryo-EM) advancements have revealed the structures of Aβ42 fibrils in the human brain, identifying two distinct fibril types: Type I, associated with sporadic AD, and Type II, linked to familial AD. Both types feature S-shaped folded protofibrils [5]. The Aβ42 fragment, primarily derived from the aforementioned processes, exhibits neurotoxicity and promotes the activation of inflammatory and oxidative cascades [6]. Therefore, strategies that prevent Aβ aggregation and boost brain antioxidant defenses offer promising therapeutic approaches to AD prevention and management.

Molecular dynamics (MD) simulations have become a pivotal computational tool in pharmacological research and are extensively employed to elucidate the dynamic mechanisms underlying drug–target interactions and molecular recognition processes [7]. By modeling atomic motions within force fields, MD enables the analysis of binding modes, conformational changes, and binding free energies between small molecules and biomacromolecules such as proteins and nucleic acids at the atomic level. This approach provides mechanistic insights into inhibitor function, stability, and specificity. Compared to static structural analyses, MD simulations capture critical residue interactions, allosteric effects, and water-mediated hydrogen bond networks during binding events, thereby significantly enhancing the depth and reliability of mechanistic studies [8]. Their unique advantages are evident in drug screening, lead compound optimization, and multi-target drug design.

*Caenorhabditis elegans*, a classical model organism, possesses a semi-transparent body and a complete intestinal system, facilitating visualization of neurons and fluorescently labeled proteins [9]. *C. elegans* offers significant benefits for neurodegenerative disease research due to its high genetic similarity to humans and its simple highly conserved nervous system, which consists of 302 neurons and around 7000 synapses [10]. Transgenic *C. elegans* models such as CL2006, CL4176, and GMC101 have been widely used in recent years to study Aβ toxicity and Tau protein abnormalities in AD. These models express human Aβ1–42 or mutant Tau proteins under muscle- or neuron-specific promoters, recapitulating key AD pathological features, including progressive paralysis, motor decline, shortened lifespan, and Aβ aggregation [11,12]. Due to their short lifecycle, low experimental cost, and comprehensive evaluative capacity, these models have become vital tools in AD research, drug screening, and the identification of novel therapeutic targets.

Traditional Chinese Medicine (TCM) and ethnomedicine offer a rich repository of natural compounds, which are useful due to their diverse range of sources and broad pharmacological activities, making them a significant treasure trove for modern drug discovery. Recent years have witnessed a surge in research focused on isolating and identifying single chemical constituents from traditional medicines with potential anti-Alzheimer’s disease (AD) activity. As a notable example in this field, the Yi ethnomedicine Tianma is derived from the dried tuber of *Gastrodia elata* Blume (Orchidaceae). In classical TCM theory, it is characterized by a neutral nature and sweet flavor, primarily acting on the Liver channel. Its documented functions include calming endogenous wind to relieve convulsions, suppressing hyperactive liver yang, and dispelling wind to unblock collaterals, which establishing it as a key therapeutic agent for dizziness and headache. Contemporary scientific investigations aim to elucidate its pharmacologically active material basis. Among them, 3,4-dihydroxybenzaldehyde (DBD), also known as protocatechuic aldehyde, is a key natural phenolic aldehyde active monomer found in *Gastrodia elata*. Its molecular structure is characterized by two adjacent phenolic hydroxyl groups (at the 3- and 4-positions) and an aldehyde group attached to the benzene ring. The ortho-dihydroxy structure confers significant electron-donating capacity, enabling effective scavenging of free radicals and chelation of metal ions and thereby inhibiting oxidative stress-mediated cellular damage [13]. Evidence indicates that DBD exhibits anti-inflammatory and antioxidant activities across various disease models. In Parkinson’s disease (PD) cell models, protocatechuic aldehyde markedly enhances cell viability, mitochondrial redox activity, and membrane potential, while reducing intracellular reactive oxygen species (ROS) levels [14]. Additionally, it restores Akt pathway activity suppressed by oxidative stress and inhibits H_2_O_2_-induced overoxidation of DJ-1 in SH-SY5Y cells [15]. The presence of two phenolic hydroxyl groups and an aldehyde group in its structure endows the molecule with the capacity to act as both a hydrogen bond donor and acceptor, coupled with moderate hydrophobicity (LogP~1.3). This unique combination of properties enables DBD to flexibly engage with biological macromolecules through multifaceted non-covalent interactions, including hydrogen bonding, hydrophobic effects, and π-π stacking. These interactions are likely provide the chemical basis for its ability to disrupt the structural stability of Aβ fibrils, thereby contributing to the inhibition of amyloid fibril formation. Resveratrol interacts with Aβ-42 dimers via π-π stacking interactions, inducing conformational changes in Aβ oligomers and attenuating their cytotoxicity [16]. Quercetin similarly inhibits Aβ fibrillogenesis by reducing β-sheet and turn structures while increasing the proportion of disordered Aβ42 dimer conformations [17]. Natural polyphenols are crucial in developing new therapeutics due to their unique phenolic ring structures, which facilitate anti-amyloidogenic activity by engaging in hydrophobic interactions and π-π stacking with aromatic residues in amyloid proteins, thus preventing further fibril aggregation [18]. These mechanistic insight provides a theoretical foundation for developing polyphenol-based neuroprotective agents.

In this study, we employ molecular dynamics simulations combined with a *C. elegans* model to investigate the protective effects of DBD against Aβ toxicity and its underlying mechanisms. Molecular dynamics simulations suggested that DBD can bind to and destabilize Aβ protofibrils. In the AD *C. elegans* model, DBD treatment led to multifaceted phenotypic improvements, including delayed paralysis progression, enhanced resistance to oxidative stress, improved locomotor behavior, and extended lifespan within the model context. To explore the mechanisms involved, we assessed relevant biochemical markers and found that DBD treatment reduced levels of reactive oxygen species (ROS) and lipofuscin, as well as Aβ deposition in the worms. Additionally, comprehensive target metabolomics and GFP reporter gene analyses reveal the mechanisms underlying DBD’s anti-AD activity. The results of this study can guide future research on the therapeutic potential of *Gastrodia elata* active compounds in AD.

## 2. Results

### 2.1. Analysis of the Protein-Ligand Interaction Complex

The binding affinity between DBD and Aβ protofibrils was assessed using compound structures from the PubChem database (https://pubchem.ncbi.nlm.nih.gov/, accessed on 2 February 2026) and the target protein structure sourced from the PDB database (https://www.rcsb.org/, accessed on 2 February 2026). The protein was prepared using PyMOL 2.1.0 by removing water molecules and small molecule ligands, followed by hydrogen addition and charge assignment using AutoDock Tools 1.5.6. The ligand and receptor were subjected to molecular docking with AutoDock Vina 2.0 integrated within PyRx software (version 1.2), calculating binding energies and generating output files. The binding affinity (kcal/mol) indicates the strength of interaction, with lower values representing more stable ligand-receptor complexes. Visualization of docking results was performed using Discovery Studio 2020 Client (https://discover.3ds.com/discovery-studio-visualizer-download, accessed on 2 February 2026) and PyMOL. Notably, the compound formed an Amide-Pi Stacked interaction with the GLY29 residue on chain B and a hydrogen bond with LYS29 on chain D, with a binding energy of −5 kcal/mol, indicating a relatively strong binding affinity.

**Figure 1:**
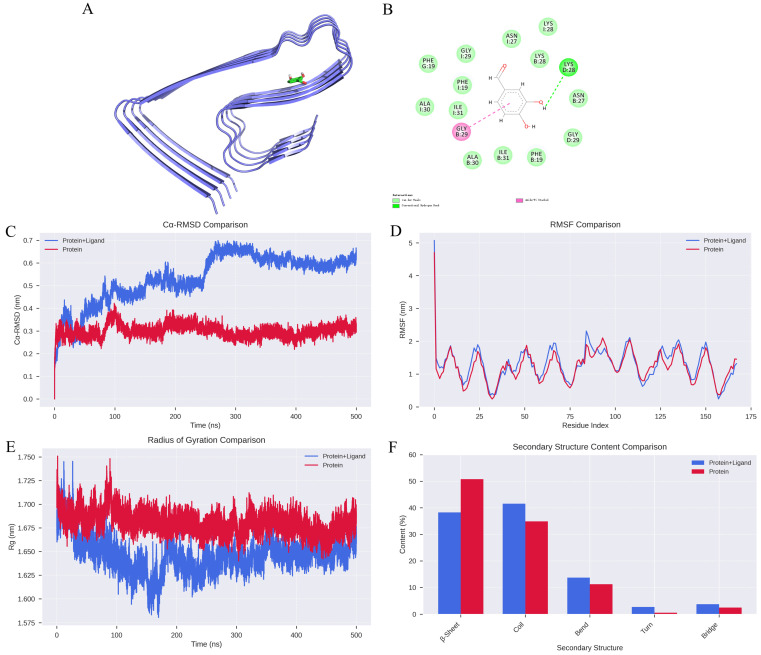
Analysis of Molecular Dynamics Simulation Results. (**A**) Structural depiction of Aβ protofibril in complex with DBD. (**B**) Hydrogen bonding interactions between DBD and Aβ protofibril. (**C**) RMSD curves for Protein and Protein + Ligand systems. (**D**) RMSF profiles for Protein and Protein + Ligand systems. (**E**) Radius of gyration (Rg) trajectories for Protein and Protein + Ligand complexes. (**F**) Changes in secondary structural content.

The impact of DBD on the stability of Aβ protofibrils was assessed through root mean square deviation (RMSD), a key indicator of protein-ligand complex stability. The results showed that the RMSD curves for the protein-ligand complexes fluctuated within approximately 0.2 nm without significant deviations, suggesting high stability of the apo protein system, whereas individual complexes exhibited notable fluctuations (Figure 1C). Root mean square fluctuation (RMSF) analysis revealed that amino acid residues in the unbound protein fluctuated within 1 nm, indicating relative stability, while complexes with DBD showed increased fluctuations (Figure 1D). The addition of DBD altered the compactness of the protofibril structure; molecular dynamics-derived radius of gyration (Rg) curves indicated larger Rg values for the complexes, implying reduced structural stability of the protofibrils (Figure 1E). Furthermore, secondary structure analysis demonstrated significant differences between the free protein and the protein-ligand complexes. In the bound state, the proportion of β-sheet content decreased from 50.81% in the unbound protein to 38.27%, suggesting that ligand binding may disrupt ordered folding regions, thereby weakening the overall rigidity. Conversely, the proportion of random coil structures increased from 34.91% to 41.58%, with slight increases in bend, turn, and bridge structures, cumulatively rising from 14.28% to 20.15% (Figure 1F). These changes reflect a shift toward a more flexible, disordered conformation, facilitating adaptation to the dynamic binding environment.

### 2.2. DBD Delays Aβ-Induced Paralysis and Determination of Optimal Concentration

**Figure 2:**
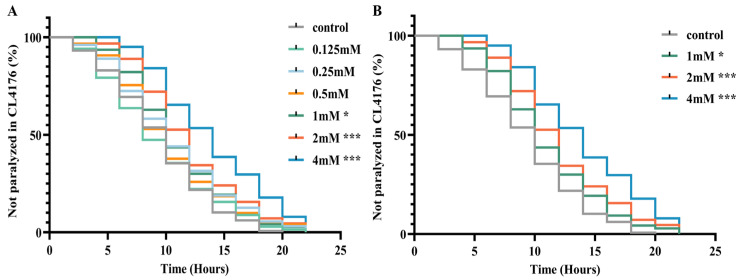
DBD delays paralysis in AD model nematodes. (**A**) Paralysis curves of CL4176 nematodes treated with various DBD concentrations, showing significant delay at 1 mM, 2 mM, and 4 mM. (**B**) Comparative analysis of paralysis efficiency between 1 mM, 2 mM, 4 mM DBD treatments and control. * *p* < 0.05, *** *p* < 0.001.

The CL4176 transgenic nematode strain serves as an Alzheimer’s disease model by conditionally expressing the Aβ42 peptide in a temperature-sensitive manner within body wall muscle cells. Elevated temperatures induce high levels of Aβ expression, increasing toxicity to muscle cells and resulting in paralysis phenotypes [19]. To assess DBD’s impact on Aβ-induced paralysis progression, nematodes underwent treatment with DBD at concentrations of 0.125, 0.25, 0.5, 1, 2, and 4 mM, followed by paralysis assays. The results demonstrated that the 1, 2, and 4 mM doses significantly delayed paralysis onset, with 2 and 4 mM exhibiting the most pronounced effects (Figure 2A,B, * *p* < 0.05, *** *p* < 0.001). Consequently, 1, 2, and 4 mM were designated as low, medium, and high concentrations for subsequent experiments. These findings indicate that DBD effectively mitigates Aβ toxicity-induced paralysis.

### 2.3. DBD Delays Nematode Aging, Enhances Motility, and Has No Significant Impact on Growth and Development

**Figure 3:**
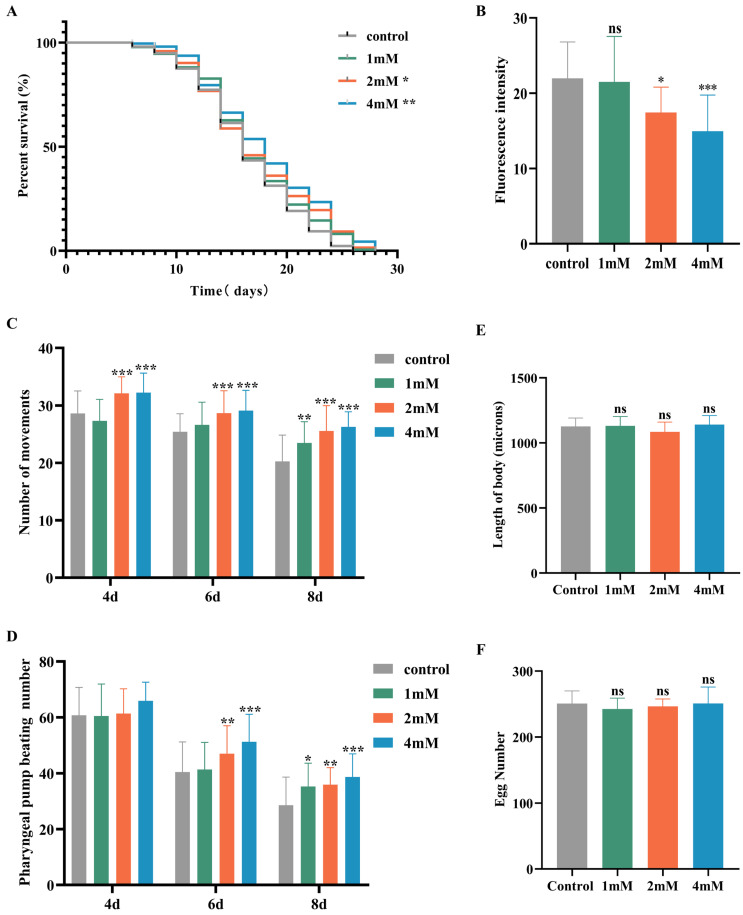

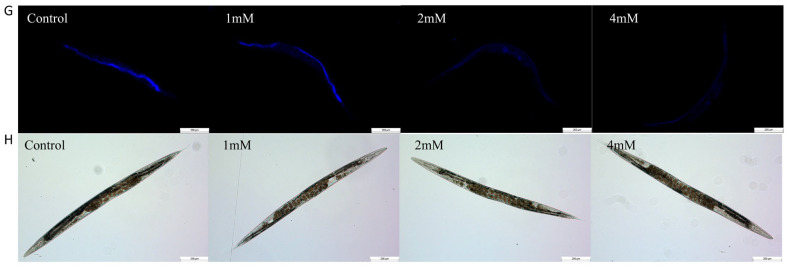
DBD extends the healthspan of nematodes. (**A**) Survival curves of nematodes exposed to DBD (1 mM, 2 mM, 4 mM). (**B**) Relative lipofuscin fluorescence intensity. (**C**) Body bending frequency of adult nematodes on days 4, 6, and 8. (**D**) Pharyngeal pumping rate on days 4, 6, and 8. (**E**) Nematode body length. (**F**) Number of eggs laid. (**G**) Lipofuscin fluorescence images of nematodes at different doses. (**H**) Body length images of nematodes at different doses. * *p* < 0.05, ** *p* < 0.01, *** *p* < 0.001. ns: not significant.

Aging significantly contributes to the advancement of Alzheimer’s disease [20]. In this study, nematodes were treated with high, medium, and low doses of DBD, along with a control group, with periodic observations of their physiological state. The maximum lifespan of the control and treatment groups was 26 and 28 days, respectively. Lipofuscin distribution in nematodes was localized near the intestine, accumulating progressively with age and producing autofluorescence [21]. The fluorescence intensity, indicative of lipofuscin accumulation and thus aging, was higher in the control group compared to the DBD-treated groups, with the high-dose group showing a significantly lower fluorescence intensity (Figure 3B, *p* < 0.001). These findings suggest that DBD reduces lipofuscin levels and delays aging in nematodes.

With increasing age, the pharyngeal pumping rate in nematodes declines, a higher pumping rate is indicative of delayed senescence. Compared to the control, no significant change in pharyngeal pumping was observed on day 4 post-treatment. On days 6 and 8, the treatment groups, especially the high-dose group, showed significantly increased pumping frequencies compared to controls (Figure 3D, *p* < 0.001). Nematode motility, a fundamental indicator of neural function and aging, was also enhanced. Data demonstrated that on days 4, 6, and 8 post-treatment, the motility of nematodes in the medium and high-dose groups was significantly improved (Figure 3C). Reproductive capacity assessments, which evaluate the potential reproductive toxicity of the compound and are correlated with lifespan and aging, showed no significant differences in progeny number between the control and treatment groups at any dose level, indicating that DBD does not impair reproductive function (Figure 3F). Furthermore, body length measurements showed no notable differences between the treated and control groups, indicating that DBD does not have toxic effects on the growth and development of nematodes (Figure 3E).

### 2.4. Effect of DBD on Thermotolerance, Oxidative Stress Resistance, and ROS Levels in C. elegans

**Figure 4:**
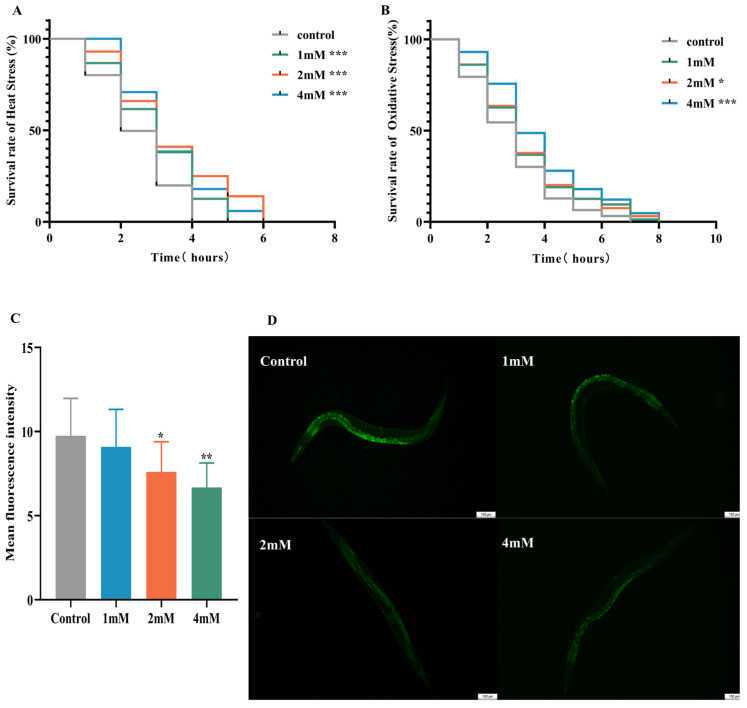
Effects of DBD on stress resistance and antioxidant capacity in worms. (**A**) Survival curve under heat stress (37 °C). (**B**) Survival curve under oxidative stress (300 μM juglone). (**C**) Quantitative analysis of ROS fluorescence intensity using ImageJ software (version 1.54). (**D**) Representative fluorescence images of ROS levels in N2 worms from the model group and groups treated with low, medium, and high doses, scale bar: 100 μm. * *p* < 0.05, ** *p* < 0.01, *** *p* < 0.001.

Elevated temperatures cause metabolic disruptions and enzyme inactivation, leading to an increase in both reactive oxygen species (ROS) and oxidative stress. This study examined the effects of drug administration on the thermotolerance of N2 worms exposed to 37 °C. After 4 h, all control worms perished, whereas worms treated with low, medium, and high doses of DBD exhibited significantly prolonged survival times (Figure 4A, *p* < 0.001). Juglone, an oxidant that induces substantial ROS generation, was employed to establish an oxidative stress model. Under oxidative stress induced by 300 μM juglone, N2 worms treated with DBD demonstrated increased survival rates, with the high-dose group showing a rightward shift in survival curves and significantly higher viability compared to controls, suggesting that DBD augments antioxidant defenses (Figure 4B). ROS free radicals are generated during oxidative injury, and their excessive accumulation disrupts redox homeostasis, leading to oxidative stress and further tissue damage [22]. In this experiment, 5 mM paraquat was used to elevate ROS levels. DCFH-DA fluorescence quantification showed a significant decrease in ROS levels in the medium- and high-dose groups (Figure 4C, *p* < 0.01), suggesting that DBD partially inhibits ROS production in worms.

### 2.5. DBD Reduces Aβ Deposition Levels in C. elegans

**Figure 5:**
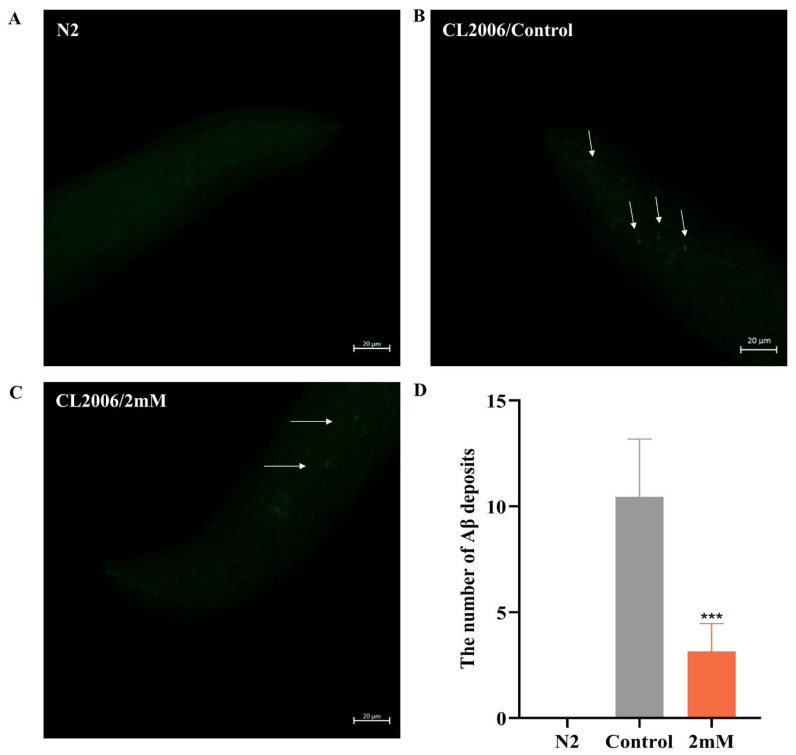
DBD decreases Aβ protein deposition in transgenic CL2006 worms. (**A**) N2 worms as negative controls showing no Aβ deposits. (**B**) Representative images of Aβ deposits in CL2006 worms. Arrows indicate representative Aβ plaques under fluorescent staining. (**C**) Images of CL2006 worms treated with DBD showing markedly reduced Aβ deposits. (**D**) Quantification of Aβ deposits per group, *** *p* < 0.001.

Early accumulation of amyloid-beta (Aβ) in the brain is a hallmark of AD [23]. In this study utilized sulfurine S dye was used for fluorescence staining to observe Aβ fibril formation and evaluate the effects of DBD on Aβ aggregation in worms. Transgenic CL2006 worms, which express Aβ peptide fragments in muscle cells leading to progressive paralysis, served as the experimental model. Fluorescent imaging of the worm head revealed no Aβ deposits in wild-type N2 controls. Compared to the control group, DBD treatment significantly reduced Aβ deposition in CL2006 worms (Figure 5, *p* < 0.001). The findings indicate that DBD treatment reduces amyloid-like deposits in transgenic worms, an effect that may be related to the inhibition of Aβ aggregation.

### 2.6. The Impact of DBD on Metabolite Abundance in CL4176

**Figure 6:**
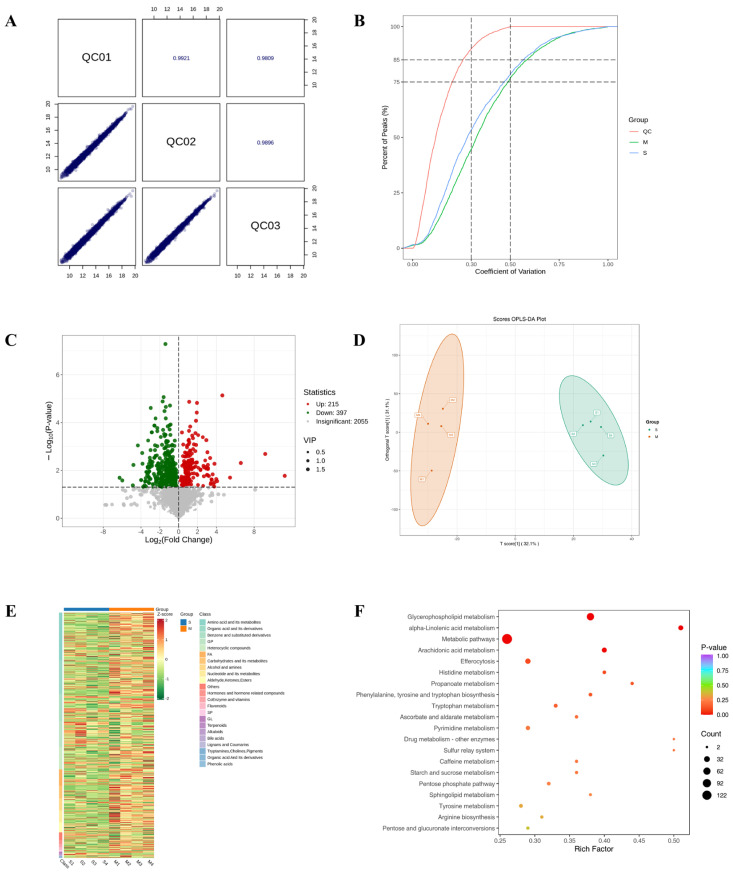
Results of TM broad-target sequencing analysis. (**A**) QC sample correlation plot. (**B**) CV distribution across groups. The vertical dashed lines at CV = 0.3 and 0.5, and the horizontal dashed lines at 75% and 85% of total features serve as reference thresholds. A higher proportion of QC sample features below these CV thresholds indicates greater experimental stability. (**C**) Volcano plot of differential metabolites. (**D**) OPLS-DA score plot. The score plot displays the first predictive component (T score[1]) against the first orthogonal component (Orthogonal T Score[1]). Percentages in parentheses indicate the explained variance for each component. (**E**) Hierarchical clustering heatmap of differential metabolites. (**F**) Pathway enrichment analysis of differential metabolites.

To elucidate the therapeutic actions of DBD, comprehensive metabolomic profiling was performed in a worm model of Alzheimer’s disease. Initially, Pearson correlation analysis of QC samples revealed correlation coefficients (r) approaching 1, indicating stable detection processes and high data quality. Furthermore, the distribution of coefficients of variation (CV) across all samples showed that over 75% of detected metabolites in QC samples had CVs below 0.3, demonstrating experimental stability (Figure 6A,B). Orthogonal Partial Least Squares Discriminant Analysis (OPLS-DA) integrates orthogonal signal correction with PLS-DA to decompose the X matrix into components related and unrelated to Y, highlighting significant intergroup differences (Figure 6C). A total of 612 differential metabolites were identified using the criteria of VIP > 1 and *p* < 0.05, comprising 215 downregulated and 397 upregulated metabolites. In order to effectively illustrate the relative variations in metabolite levels, a heatmap was created to show the hierarchical clustering of differential metabolites (Figure 6D,E). The initial categorization of these metabolites showed correlations with amino acids and their derivatives, organic acids along with their derivatives, and nucleotides and their byproducts, as well as coenzymes, vitamins, alkaloids, flavonoids, and terpenoids (Figure 6F).

### 2.7. The Effect of DBD on the Expression of daf-16, skn-1 sod-3 and gst-4 in Worms

**Figure 7:**
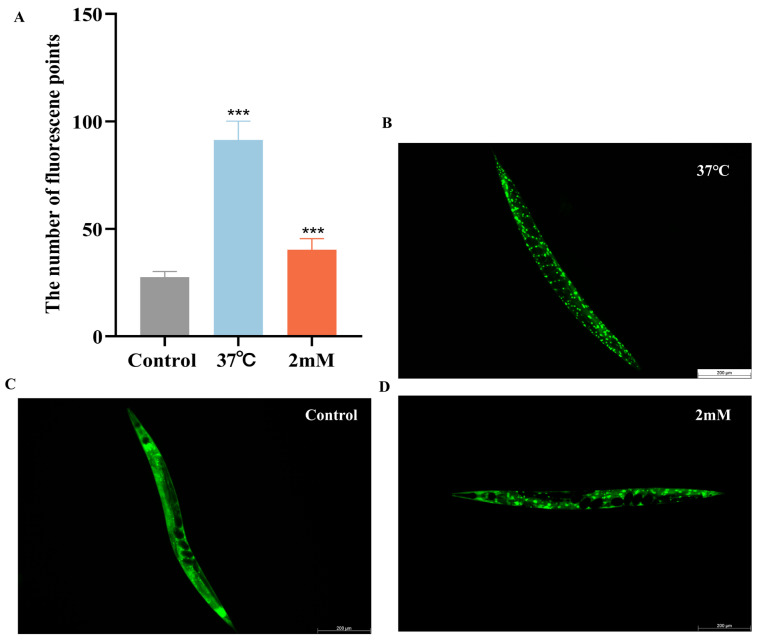

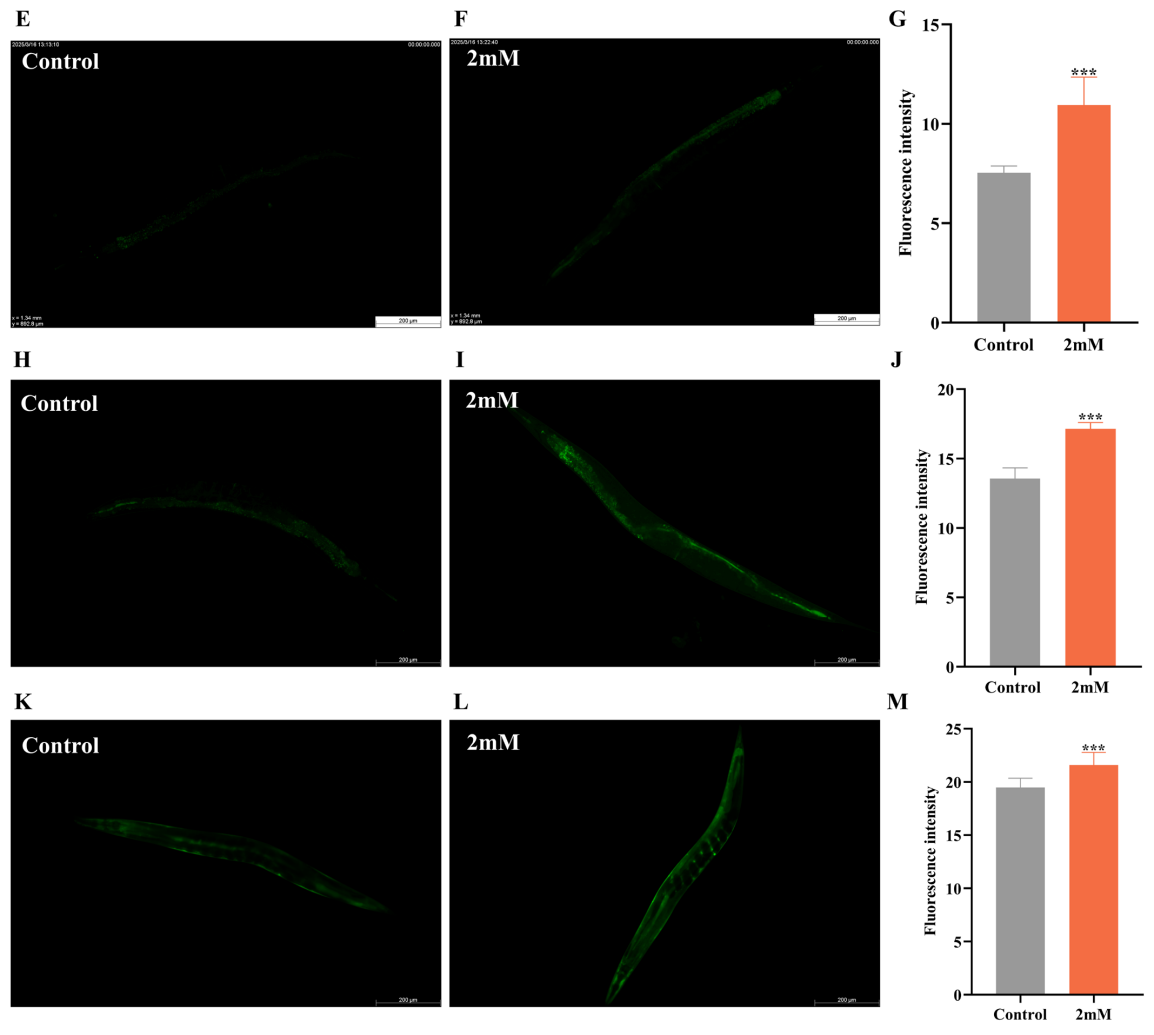
DBD induces the expression of daf-16/FOXO, skn-1/nrf2, sod-3 and gst-4. Representative images of TJ356 worms: (**A**) Number of fluorescence points in each group. (**B**) Nuclear localization in positive group. (**C**) Control group. (**D**) nuclear translocation in DBD-treated group. Representative images of LD1 worms: (**E**) Control group. (**F**) DBD-treated group. (**G**) Relative fluorescence intensity of SKN-1::GFP in LD1 worms. Representative images of CF1553 worms: (**H**) Control group. (**I**) DBD-treated group. (**J**) Relative fluorescence intensity of SOD-3::GFP in CF1553 worms. Representative images of CL2166 worms: (**K**) DBD-treated group. (**L**) Control group. (**M**) Relative fluorescence intensity of GST-4::GFP in CL2166 worms. *** *p* < 0.001.

It has been reported that daf-16, a FOXO transcription factor in *C. elegans*, is homologous to mammalian FOXO proteins and influences lifespan and stress resistance [24]. To investigate whether the lifespan extension induced by DBD is associated with daf-16 activity, we examined the effect of DBD on daf-16 nuclear localization using the TJ356 strain. The results showed that DBD treatment significantly promoted daf-16 nuclear translocation (Figure 7A–D, *p* < 0.001). Compared to the control group, the DBD-treated group exhibited a significant increase in SOD-3::GFP expression (Figure 7H–J). Additionally, skn-1, the *C. elegans* homolog of Nrf2, is known to modulate lifespan primarily through oxidative stress responses [25]. Given that DBD significantly enhanced oxidative stress tolerance, we further explored the role of skn-1 in this process. The study showed that DBD treatment led to a notable increase in skn-1 expression (Figure 7E–G) and GFP fluorescence in the GST-4::GFP strain (Figure 7K–M). These results indicate that DBD treatment is associated with enhanced skn-1 pathway activity, which may subsequently contribute to the regulation of antioxidant defenses. In summary, the lifespan extension and improved stress resistance induced by DBD are consistent with enhanced activity of both daf-16 and skn-1.

## 3. Discussion

Alzheimer’s disease (AD) is a major neurodegenerative disorder with significant global public health impacts. Its pathogenesis is complex, and therapies capable of effectively halting or reversing disease progression are currently lacking [26]. Among various pathological hypotheses, the β-amyloid (Aβ) cascade hypothesis and oxidative stress theory are considered intertwined core processes. Oxidative stress is not only an early key event in AD but also forms a vicious cycle with Aβ toxicity, jointly driving neuronal damage and cognitive decline [27]. Consequently, exploring compounds that can simultaneously intervene in Aβ toxicity and oxidative stress has become a crucial strategy in current AD drug development [22,28]. *Gastrodia elata*, a traditional Chinese herb, has shown potential in neuroprotection through its active components, such as 4-hydroxybenzyl alcohol, which has been reported to ameliorate Aβ-induced toxicity [29]. This study focuses on DBD, another phenolic component from *Gastrodia elata*, aiming to systematically evaluate its anti-AD potential and preliminarily explore its mechanism of action [30].

Computational simulations in this study suggested that DBD could bind to Aβ fibrils and might thereby reduce their structural stability, supporting its potential to interfere with Aβ aggregation. Subsequently, in *C. elegans* AD models expressing human Aβ1-42 (strains CL4176), DBD treatment significantly improved the paralysis phenotype induced by Aβ expression, extended lifespan, and showed no reproductive toxicity at effective concentrations. Phenotypic improvements were accompanied by enhanced motility and increased resistance to oxidative stress (heat and juglone). Further biochemical analyses demonstrated that DBD effectively reduced levels of reactive oxygen species (ROS) and lipofuscin in the worms. Notably, thioflavin S staining revealed a significant reduction in amyloid-like deposits in DBD-treated worms. It is important to emphasize that the *C. elegans* model used in this study induces toxicity through ectopic expression of Aβ in body wall muscles. Its core pathology aligns more closely with generalized cellular stress and mitochondrial dysfunction triggered by misfolded protein aggregation, rather than specifically mimicking intraneuronal Aβ deposition as seen in AD. Therefore, the alleviation of paralysis and reduction in deposits by DBD should first be interpreted as an overall amelioration of proteotoxic stress. While molecular docking and dynamics simulations provide structural-level support for DBD’s potential interference with Aβ fibrillization, the reduction in thioflavin S staining is consistent with a decrease in amyloid-like deposition. This outcome may stem from the direct inhibition of the Aβ aggregation process, or it could be achieved through enhancing cellular autophagy clearance capacity and improving intracellular homeostasis [31]. Further mechanistic studies require more direct experimental investigations to delve deeper into these possibilities.

To understand DBD’s effects at a systems level, we performed untargeted metabolomic analysis on DBD-treated worms. A total of 612 differential metabolites (VIP > 1, *p* < 0.05) were identified. The alterations in these metabolites indicate a remodeling of the metabolic network in DBD-treated worms [32,33]. Among the significantly upregulated metabolites, we identified several substances previously reported in the literature to exert neuroprotective activities, primarily associated with antioxidant and anti-Aβ toxicity mechanisms. For instance, vanillic acid (VA) significantly enhances antioxidant capacity by upregulating HO-1 expression through the activation of the Akt/GSK-3β/Nrf2 pathway [34]. Verbascoside mitigates neuroinflammation and apoptosis by downregulating pro-inflammatory cytokines IL-1β, IL-6, and TNF-α, while upregulating the PI3K/AKT pathway [35]. Docosahexaenoic acid (DHA) and its metabolites not only reduce Aβ42 production but also improve synaptic plasticity through antioxidative effects [36]. Rosmarinic acid (RA) exhibits dual regulatory roles by intervening in amyloidogenic pathways and decreasing oxidative stress markers MDA and NOx while increasing SOD activity [37]. Metabolites such as uric acid derivatives improve tau hyperphosphorylation and Aβ toxicity via PI3K/Akt/GSK-3β pathways [38]. Gut microbiota-derived metabolites such as END and ENL from SDG inhibit Aβ deposition via the GPER-CREB/BDNF pathway, whereas L-theanine provides neuroprotection by influencing SIRT1 and AGEs/RAGE signaling pathways [39,40]. The upregulation of these endogenous protective metabolites strongly suggests that DBD may not function merely as a single direct-acting molecule but rather as a “metabolic switch” activating or promoting the host’s intrinsic neuroprotective pathways. Concurrently, levels of several metabolites associated with disease progression were downregulated after DBD treatment. For example, the accumulation of succinyl-CoA and its derivatives can trigger acute brain injury, and putrescine produced by astrocytic urea metabolism is closely linked to memory impairment [41]. D-ribose, due to its strong glycation ability, is a significant pathogenic factor that accelerates the abnormal modifications of tau and Aβ proteins through the formation of advanced glycation end products (AGEs) [42]. Elevated levels of metabolites such as ergothioneine and 1,7-dimethyluric acid are positively correlated with AD risk [43], while oxidation of methionine impairs clustering function, further aggravating Aβ toxicity [44]. The overall metabolomic profile indicates that DBD intervention shifts the worm’s metabolic state toward one more conducive to resisting proteotoxicity and oxidative damage.

Building on the antioxidant stress direction suggested by metabolomics, we further validated DBD’s effects on key stress-response pathways at the molecular level. The results showed that DBD significantly promoted the nuclear translocation of the transcription factor DAF-16 and enhanced the expression of its downstream target gene sod-3. Concurrently, DBD activated another crucial transcription factor, SKN-1, and its downstream target gene gst-4. DAF-16/FOXO and SKN-1/Nrf2 are evolutionarily highly conserved central pathways regulating lifespan, oxidative stress response, and autophagy. This study clearly establishes a strong correlation between DBD treatment and the activation of these two key pathways. Their coordinated activation provides a plausible explanation for the observed antioxidant, anti-aging, and anti-proteotoxic phenotypes, suggesting that DBD may function by activating an intrinsic cellular defense network mediated by DAF-16 and SKN-1. However, it is essential to note that the current evidence establishes correlation. To establish the functional necessity of DAF-16 and SKN-1 in mediating DBD’s protective effects, future loss-of-function experiments using genetic tools are required. If DBD’s protective effects are abolished in daf-16 or skn-1 loss-of-function backgrounds, it would provide the most direct evidence for the mechanism.

## 4. Materials and Methods

### 4.1. Reagents and C. elegans Strains

The compound DBD was acquired from Chengdu Alpha Biotechnology Co., Ltd. (Chengdu, China). The compound was precisely weighed with an ME104 electronic balance, dissolved in deionized water to a 10 mM concentration, sterilized by filtration, and stored at −20 °C as a stock solution. Serial dilutions of the DBD stock solution were prepared using an OP50 bacterial culture to achieve target concentrations (0.125, 0.25, 0.5, 1, 2, and 4 mM) and were subsequently mixed into NGM agar plates. Control groups were administered a comparable volume of bacterial suspension devoid of the drug. The optimal drug concentration was determined via paralysis assays and subsequently used in all further experiments.

This study utilized various transgenic *C. elegans* strains, including CL4176, CL2006, TJ356, LD1, CF1553, CL2166, and the wild-type N2 strain. The transgenic strains utilized in this study were selected to model Aβ pathology and monitor protective stress pathways. Aβ expression was achieved using CL4176 (dvIs27 [myo-3p::A-β(1–42)::let-851 3′UTR + rol-6(su1006)]X) and CL2006 (dvIs2 [pCL12 (unc-54::human Aβ(1–42) minigene) + rol-6(su1006)]). Concurrently, key defense pathways were tracked with the following reporters: TJ356 (zIs356 [daf-16p::daf-16a/b::GFP + rol-6(su1006)]) for DAF-16, LD1 (ldIs7 [skn-1b/c::GFP + rol-6(su1006)]) for SKN-1, CF1553 (muIs84 [pAD76 (sod-3::GFP) + rol-6(su1006)]) for SOD-3, and CL2166 (dvIs19 [pAF15 (gst-4p::GFP::NLS)] III) for GST-4. Strains were sourced from the Caenorhabditis Genetics Center (CGC) at the University of Minnesota and cultured on Escherichia coli OP50. Cultures were incubated at 20 °C, with CL4176 maintained at 16 °C.

### 4.2. Molecular Docking and Molecular Dynamics Simulations

Native fibrils were isolated via cryo-electron microscopy (PDB ID: 5OQV). The Aβ42 tetramer was selected as the protofibril model, given its characterization as the minimal nucleating fibril of Aβ [45]. AutoDock Vina facilitated molecular docking with a grid box of 262,798 × 112 × 80 Å. PyMOL and LigPlot+ (version 2.2) were employed to visualize docking conformations and identify interactions between Aβ42 protofibrils and the DBD domain.

Molecular dynamics simulations were performed using GROMACS and the AMBER99SB-ILDN force field to analyze the conformational dynamics of Aβ42 protofibrils (control) and Aβ42 protofibril-DBD complexes (treatment) [46]. Force field parameters for DBD were generated via the Automated Topology Builder (ATB). The protofibril was centered within a cubic simulation box, maintaining a minimum solute-box edge distance of 1.0 nm. To simulate physiological pH conditions and maintain overall neutrality, 0.15 M NaCl was added to each system. Post energy minimization, the solvent environment comprised 19,677 and 19,664 water molecules modeled with TIP3P, surrounding the control DBD system. The treatment of long-range electrostatic interactions was conducted with the Particle Mesh Ewald (PME) algorithm. After steepest descent energy minimization, systems underwent equilibration in the NVT ensemble for 500 ps, followed by the NPT ensemble for another 500 ps, using the Parrinello-Rahman barostat and velocity-rescale thermostat at 1 bar and 310 K. Subsequently, production MD simulations were performed for 500 ns under these conditions [47].

### 4.3. Paralyzing Assay

In the CL4176 nematode model, human Aβ1–42 is expressed in body wall muscle cells, regulated by the myosin promoter. At 16 °C, the smg-1 pathway remains active, recognizing and degrading Aβ transcripts containing erroneous 3’ untranslated regions. At the L3 stage, increasing the temperature to 25 °C deactivates the smg-1 pathway, allowing Aβ1–42 mRNA expression and aggregation of Aβ oligomers in muscle cells, resulting in progressive paralysis phenotypes [48]. To induce Aβ1–42 expression, CL4176 worms were shifted from 15 °C to 25 °C starting at the L3 stage. This timing ensured developmental synchrony while providing a sufficient induction window for the paralytic phenotype to become fully penetrant during adulthood. Synchronized L1-stage CL4176 worms were placed on NGM plates, with or without pharmacological agents, at a density of approximately 30–50 worms per plate, using three parallel plates for each group. The temperature was maintained at 16 °C for 36 h, and then raised to 25 °C for the next 24 h. Worm paralysis was monitored and recorded every 2 h until complete paralysis was observed.

### 4.4. Lipofuscin Quantification

L4-stage *C. elegans* of the N2 strain were synchronized and placed on NGM plates containing varying drug concentrations: high, medium, low, or none. After being cultured for 10 days at 20 °C, the worms were collected with PBS immobilized with 0.2% levamisole (Sichuan Microbiological Biotechnology, Chengdu, China) and examined under a fluorescence microscope to observe spontaneous lipofuscin autofluorescence. ImageJ software was utilized to measure fluorescence intensity. The experiment was conducted independently three times, with each group consisting of a minimum of 20 worms.

### 4.5. Lifespan Assay

L4-stage *C. elegans* of the CL4176 strain were synchronized and placed on NGM plates with varying drug concentrations (1, 2, and 4 mM) or drug-free controls, all containing 12 μM Fudr to prevent progeny production. The worms were maintained at 16 °C, designated as Day 0 of survival. Every two days, the number of deceased worms was documented, and the surviving worms were moved to new plates with the same drug concentration for ongoing observation until all had perished.

### 4.6. Motility and Pharyngeal Pumping Assays

Synchronized L3-stage N2 worms were placed on NGM plates with varying drug doses (high, medium, low, or none) and kept at 20 °C, marking the start as day 0. To assess locomotor activity, worms were transferred to fresh plates with 0.1 mL M9 buffer on days 4, 6, and 8. The number of body bends (sinusoidal movements) that each worm completed in a 30-s interval was quantified, with 10 worms assayed per plate. A sinusoidal movement consists of a head swing combined with a body bend.

Following synchronization at the L3 stage, worms were maintained at 20 °C on plates containing varying drug concentrations or no drug. Pharyngeal pumping was documented under a dissecting microscope at specified time points (days 4, 6, and 8). The pumping frequency was determined by counting the pharyngeal contractions within 30 s for each of the 20 worms per group.

### 4.7. Reproductive Capacity and Body Length Assay

Total progeny were counted to assess the effects of drugs on reproduction [49]. The reproductive capacity of synchronized L4-stage CL4176 worms was assessed using a daily transfer protocol. Briefly, two worms were placed on NGM plates with varying drug doses (high, medium, low) or vehicle control at 16 °C, and transferred daily from day 1 until the end of the egg-laying period. Egg plates were incubated at 16 °C, with larval hatching counted on day 3, followed by daily and cumulative egg counts on subsequent days.

L4-stage CL4176 worms were placed on plates with varying drug concentrations (high, medium, low, or none), each supplemented with 12 μM Fudr to prevent egg-laying, for body length measurement. Worms were collected in PBS after 2 days, anesthetized with 0.2% levamisole, and imaged using a fluorescence microscope. At least 20 worms per group were measured for body length using ImageJ software.

### 4.8. Stress Resistance Assays

Heat Stress: Following the placement of synchronized L1 wild-type N2 worms on drug-containing or control NGM plates (three replicates per group, 30 worms each), the cultures were maintained at 20 °C for two days prior to a temperature upshift to 37 °C, where their survival was observed hourly until mortality was complete. The experiment was conducted independently on three separate occasions.

Oxidative Stress: L1-synchronized N2 worms were cultured at 20 °C on plates with different drug concentrations until reaching the L4 stage, after which they were transferred to NGM plates with 300 μM juglone to induce oxidative stress. Each group consisted of three plates with approximately 30 worms each. Survival was assessed hourly until all worms had died. Death was defined by complete body stiffening and unresponsiveness to light and mild vibrations. The experiment was conducted independently on three separate occasions.

### 4.9. ROS Detection

Synchronized L1-stage N2 nematodes were placed on NGM plates with varying drug concentrations high, medium, low, and none at approximately 50 worms per plate. The organisms were grown at 20 °C for 2.5 d. Subsequently, a 5 mM paraquat solution was added to the NGM plates. After a treatment period of 4 h, the worms were gathered and rinsed three times with PBS buffer, with the supernatant being discarded each time. Each sample was treated with 50 μL of 10 μM DCFH-DA solution and incubated at 37 °C for 30 min. Subsequently, the samples were washed three times with PBS. Finally, a 30 μL worm suspension was placed on a glass slide, covered with a coverslip, and examined using a fluorescence microscope. Parameters were adjusted, images were saved, and fluorescence intensity was measured using ImageJ.

### 4.10. Aβ Fibril Deposition Assay

L1-synchronized CL2006 nematodes were cultured on NGM plates with bacterial suspension at 20 °C until reaching the L4 stage. They were then moved to NGM plates with a high, medium or low doses of the drug, or no drug, with three parallel plates per group, each containing about 50 worms. Worms were harvested into Eppendorf (EP) tubes with M9 buffer after two days of cultivation, centrifuged, and repeatedly washed to remove bacteria. Worms were preserved in 1 mL of 4% paraformaldehyde at 4 °C for 24 h. The fixative solution was discarded, and 1 mL of permeabilization solution was added, followed by incubation at 37 °C for 24 h. Following three TBST washes, worms were stained with fluorescent dye thioflavin S at room temperature for 2 min and subsequently decolorized twice with 50% ethanol for 2 min each. Imaging was performed using a confocal laser microscope: worm suspensions were placed on slides and coverslipped, parameters were adjusted, and images were saved, focusing on the head region, specifically the anterior pharynx, to observe Aβ protein deposition.

### 4.11. Comprehensive TM Analysis

Synchronized L1-stage CL4176 nematodes were cultured on NGM plates with or without 4 mM drug at 16 °C for 48 h, followed by a temperature shift to 25 °C for approximately 30 h. The worms were then harvested in M9 buffer, washed 2–3 times with sterile water, snap-frozen in liquid nitrogen, and stored at −80 °C for subsequent sequencing. For metabolite extraction, thawed samples were homogenized in a ball mill with stainless steel beads at 30 Hz and extracted with ice-cold 70% aqueous methanol at 4 °C prior to analysis. Non-targeted metabolomics was initially conducted to identify metabolites, which were subsequently matched with the Metware Internal Metabolite Database (MWDB) for comprehensive TM analysis. Metabolomic differences were assessed using ultra-high-performance liquid chromatography-tandem mass spectrometry (UPLC-MS/MS) alongside internal databases and multivariate statistical analysis. We acquired untargeted metabolomic data using on an UPLC-TripleTOF 6600 QTOF system (AB SCIEX, Framingham, MA, USA) and targeted data using on an UPLC-Q-Trap system. A pooled QC sample was injected periodically to ensure data quality. Compound annotation was achieved by comparing the acquired MS/MS spectra and retention times from the LC-QTOF-MS/MS analyses against integrated commercial (MWDB and its AI library) and public databases (Metlin, HMDB, KEGG). Metabolites were considered differential if they had a VIP score greater than 1 and a *p*-value less than 0.05.

### 4.12. Expression of DAF-16/FOXO, SKN-1/NRF2, GST-4, and SOD-3 in Transgenic Strains Carrying GFP Reporters

L1-synchronized worms were maintained under drug treatment or control conditions on NGM plates over a 72-h period. Worms were collected with PBS and washed three times, the supernatant was discarded, and worms were anesthetized with levamisole. They were then mounted on slides for imaging. Fluorescent images were acquired using a fluorescence microscope and analyzed with ImageJ after subtracting background signals. For the TJ356 strain, worms were incubated in a blank medium for 72 h and subsequently subjected to a 30-min heat shock at 37 °C as a positive control. The GFP fluorescence intensity of skn-1 in the intestinal region of pharyngeal-labeled worms was assessed, and daf-16 nuclear localization was quantified by counting fluorescent puncta per worm [50]. The whole-body fluorescence intensity of SOD-3::GFP and GST-4::GFP was quantified in the CF1553 and CL2166 strains, employing three independent replicates with a minimum of 10 worms per group.

### 4.13. Statistical Analysis

We employed the following analytical protocols: data were analyzed using GraphPad Prism 8.0 and are presented as mean ± SD (*n* = 3). Group comparisons were performed by *t*-test or one-way ANOVA; survival analysis was carried out using the log-rank test. Statistical significance (*p* < 0.05) is indicated as * *p* < 0.05, ** *p* < 0.01, and *** *p* < 0.001.

## 5. Conclusions

In summary, this study provides the first systematic evidence demonstrating that DBD, a phenolic compound derived from *Gastrodia elata*, exhibits significant protective effects against Aβ-induced toxicity in a *C. elegans* model. DBD treatment markedly improved core Alzheimer’s disease-related phenotypes, including paralysis, reduced motility, and shortened lifespan, while concurrently reducing amyloid-like deposition and alleviating oxidative stress. The protective effects are likely mediated through a multi-layered mechanism: DBD directly or indirectly reduces final amyloid-like deposits while systemically modulating the host metabolomic profile, promoting the upregulation of endogenous neuroprotective metabolites and the downregulation of pro-damage metabolites. A more critical mechanistic insight is that DBD’s efficacy is strongly associated with the coordinated activation of two evolutionarily conserved cytoprotective transcription factors—DAF-16/FOXO and SKN-1/Nrf2. Although the muscle-specific Aβ expression model used in this study primarily reflects generalized cellular proteotoxicity rather than neuron-specific AD pathology, it serves as a robust and relevant system for screening compounds that enhance fundamental cellular resilience. The metabolomic shift toward a neuroprotective profile further supports the notion of a systems-level intervention. Regarding future research directions, the precise molecular target of DBD and the functional necessity of DAF-16 and SKN-1 activation for its protective effects require further validation through genetic loss-of-function experiments using this model. In addition, based on the lead compounds we have found so far, we can systematically study the structure–activity relationship by introducing different functional groups to optimize their binding affinity with Aβ targets or explore the potential role of their analogues in key pathological targets. More importantly, extending this study to neuronal cell culture models and AD transgenic mouse models is essential in order to confirm DBD’s translational potential and neuron-specific efficacy. Nevertheless, the current findings establish DBD as a promising lead compound and highlight the feasibility of therapeutic strategies that enhance endogenous cellular defense pathways to combat protein-misfolding diseases such as Alzheimer’s disease.

## Data Availability

The raw data supporting the conclusions of this article will be madeavailable by the authors on request. The metabolomics data presented in the study are openlyavailable in Metabolights at accession number MTBLS13568.
